# Prevalence of High-Risk Human Papillomavirus Genotypes among Young Women in Puerto Rico; a retrospective longitudinal study

**DOI:** 10.21203/rs.3.rs-3591893/v1

**Published:** 2023-11-30

**Authors:** Yaritza León García, Lynnette A. Ruiz, William A. Calo, Susan T. Vadaparampil, Adalberto Mendoza, Rosa Vélez Cintrón

**Affiliations:** Southern Pathology Services Inc; Ponce Health Sciences University-Ponce Research Institute; Penn State College of Medicine; Moffitt Cancer Center; Southern Pathology Services Inc; Southern Pathology Services Inc

**Keywords:** Human Papillomavirus (HPV), high-risk HPV, genotypes, Hispanic, cervical cancer

## Abstract

**Background:**

Human Papillomavirus (HPV) is the most common sexually transmitted infection. High-risk HPV types are the main cause of cervical cancer. Annually, cervical cancer is among the top 10 cancers in Puerto Rican women, with 22% of these cases ending in death. The purpose of this study was to establish the prevalence of high-risk HPV genotypes in a large cohort of young women living in Puerto Rico.

**Methods:**

A retrospective longitudinal analysis was performed with a sample of 5,749 HPV results obtained from a clinical database of women ages 21 to 29 from 2014–2016.

**Results:**

Outcomes indicate that among those with a positive HPV result, about one-third (35.2%) had a high-risk HPV infection. Women between the ages of 21 to 23 showed the highest prevalence (40.6%) of high-risk HPV. Among genotypes HPV 16 and 18, genotype 16 was the most prevalent. Interestingly, 85.4% of results were positive for other high-risk HPV types other than 16 or 18. Of the 458 women who had at least two tests completed, 217 had an initial positive result for HPV and only 108 (49.7%) resolved the infection.

**Conclusions:**

This study confirms the high prevalence of several genotypes of high-risk HPV in young women in a large Puerto Rican sample.

## 1. Introduction

Cervical cancer is the fourth most common cancer in women [1, 2]. Each year, in the United States, nearly 13,000 develop cervical cancer and approximately more than 4,000 of these die from their disease [3, 4]. Cervical cancer is one of the top 10 cancers among Puerto Rican women [5]. In Puerto Rico, approximately 250 women develop cervical cancer annually; of those, 22% eventually succumb to the disease [6].

Pap testing is the cornerstone of one of the most successful cancer prevention programs [7]. With the discovery that Human Papillomavirus (HPV) causes nearly all cervical cancers, and the advent of HPV testing, newer screening and abnormal test management guidelines incorporate both Pap and HPV test results [8, 9]. Of the more than 100 strains of HPV, about 30 are oncogenic [10] while the remainder are low risk [11]. The high-risk HPV genotypes (16, 18, 31, 33, 35, 39, 45, 51, 52, 56, 58, 59, 66, and 68) are strongly linked to high-grade lesions [12], with genotypes 16 and 18 causing approximately 70% of all cervical cancers [13].

The HPV virus is very common; at least four out of five women will be infected over the course of their reproductive years [14]. Certain factors, specifically becoming sexually active at an early age, can overall increase the probability of HPV infection and increase the likelihood of a high-risk genotype infection. Epidemiological data showed that U.S. women aged 20 to 24 years old had the highest prevalence of HPV (44.8%); members of this population also had the greatest number of genotypically high-risk HPV infections [14]. Evidence shows that high-risk recurrent, persistent HPV infections have the potential to develop into cancer [15]. The information generated by this and similar studies is necessary for the improvement of cervical cancer prevention and treatment techniques for young women who are at high risk of development [16]. In Puerto Rico, there is very limited data regarding the prevalence of high-risk HPV infection and its association with premalignant and malignant lesions in women aged 21 to 29 years old [16].

The purpose of this study was to ascertain the prevalence of high-risk HPV in a sample of young adult women (21 to 29 years old) living in Puerto Rico. This data will shed light on the prevalence and persistence of HPV in the studied population. Given the rapidly evolving policy and practice changes related to cervical cancer prevention and early detection, including the 2018 mandatory school entry requirement in Puerto Rico [16] as well as the American Society of Cancer (ASC) [17] and American Society for Colposcopy and Cervical Pathology (ASCCP’s) [18] changes to cervical cancer screening and management guidelines, this study provides important baseline data to assess the impact of these changes.

## 2. Methods

### 2.1 Study design

This retrospective, longitudinal study used data of patients from Southern Pathology Services, Inc. (SPS), in Ponce, Puerto Rico. The SPS reference laboratory is a clinical pathology test center that analyzes cervical cytology samples from the entire island. The de-identified data utilized for the study included high-risk HPV results (considered to be oncogenic) as well as the genotyping for HPV subtypes 16 and/or 18, and other genotypes (31, 33, 35, 39, 45, 51, 52, 56, 58, 59, 66, and 68) of each sample from 2014 to 2016. This study was approved by the University of Puerto Rico, Medical Sciences Campus (UPR-MSC), Institutional Review Board (IRB) as an exempt protocol (#B1390117).

### 2.2 Sample

All women aged 21 to 29 years old with at least one HPV high-risk type (16, 18, 31, 33, 35, 39, 45, 51, 52, 56, 58, 59, 66, and 68) result in the SPS laboratory information system during the study period (2014 to 2016) were included. A total of 5,749 women (1,456 from 2014, 2,029 from 2015, and 2,264 from 2016) of 34,664 cases were selected. An HPV positive case was defined as a female who tested positive for HPV by RT-PCR at least one time during the study period. For women with more than one HPV result during the same year, only the first result was considered. Women with an undetermined HPV result (i.e., invalid results due to interference in the PCR reaction) were excluded from analysis.

### 2.3 Data Analysis

The data was selected and extracted by the pathology laboratory personnel using the Brio Query 6.6 software and then transferred to Microsoft Excel for statistical analysis. The dataset included demographic information, such as age and the location of the gynecological facility where the cervical sample was collected, and the year when the test was performed (data not shown). The following laboratory results were available: HPV-16, HPV-18, and other high-risk HPV genotypes (31, 33, 35, 39, 45, 51, 52, 56, 58, 59, 66, and 68). The database was de-identified by the pathology laboratory personnel prior to the transfer to the investigator.

Frequency distributions and simple descriptive statistics were used to describe data. The team estimated the prevalence of HPV (e.g., high risk, infection load, by age group) and the distribution of the genotypes HPV-16 and HPV-18 (including co-infections and in what age groups they appeared). For those with more than one test result between 2014 and 2016, the clearance of the virus was evaluated. After an initial positive result from a previous year from the same period, the clearance of HPV was defined as a negative result.

## 3. Results

**Figure 1:**
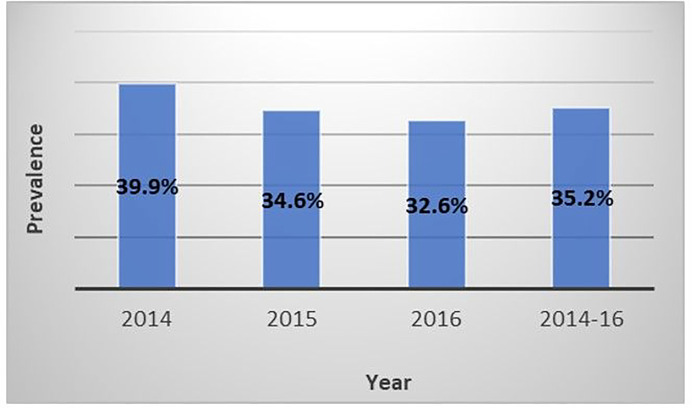
HPV prevalence of infection by year Women were considered seropositive for HPV if tested positive for at least one of the following High Risk HPV Types: 16, 18, 31, 33, 35, 39, 45, 51, 52, 56, 58, 59, 66, or 68 during the study period. 5,749 HPV results were analyzed from 2014 to 2016. The distribution of cases was the following: 1,456, 2,029, and 2,264 for the years 2014, 2015, and 2016 respectively.

In 2014, almost 40% of women in the sample were seropositive for at least one of the high-risk HPV types tested (Fig. 1). The prevalence of seropositive women was 34.6% and 32.6% in 2015 and 2016, respectively. Overall, from 2014 to 2016, 35.2% of women were seropositive for any of the high-risk HPV types evaluated. During 2014–2016, type-specific seroprevalence for high-risk HPV types was 10% for HPV 16, 3.8% for HPV 18, and < 1% for types 16 and 18 combined (Table 1). The seroprevalence for at least one of the other high-risk HPV types (non 16 and non 18) was 85.4%.

**Figure 2:**
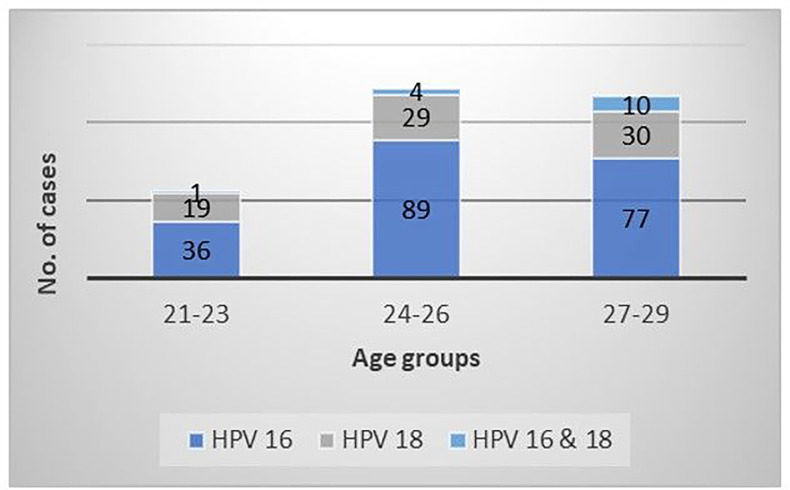
HPV Genotype 16 & 18 prevalence by age The prevalence of HPV 16, HPV 18, or a co-infection with HPV 16 and 18 subtypes was analyzed from the total of high-risk HPV positive cases found in the different groups of age included (21–23, 24–26 and 27–29 years old).

Seroprevalence of any high-risk HPV type was highest in the 21–23-year-old age group (40.5%), followed by the 24–26-year-old (35.1%) and 27–29-year-old (30.5%) groups. In the youngest age group (21–21), 64% (n = 36) of women were seropositive for HPV 16 and 34% (n = 19) for HPV 18. Seventy-three percent (n = 89) of women in the 24–26-year-old age group were seropositive for HPV 16 and 34% (n = 29) for HPV 18 (Fig. 2). In the 27–29-year-old group, 66% (n = 77) and 26% (n = 30) of women were seropositive for HPV 16 and HPV 18, respectively.

Among a subsample of 458 women with more than one HPV test completed between 2014 and 2016, 419 women had HPV test results completed in two years and 39 women had test results completed each of the three study years (Table 2). Among those with at least two tests, 196 women initially tested positive for HPV, of which 51% (n = 100) had a second positive test the next year. The other 49% percent (n = 96) of the cases with an initial positive result cleared the virus, showing a negative HPV result in the second test. Of the 223 women with an initial negative test for HPV, 14.3% (n = 32) showed a positive result the next year. Of the women who were tested three times over the course of the study period (n = 39; 2014–2016), 21 (53%) were found to have the HPV infection at their first testing; of these, seven (17.9%) experienced a clearance of their infection by the time of their second test and five (12.9%) by the time of their third test. The remaining nine (23.0%) women were HPV positive for all three years.

## 4. Discussion

By performing a descriptive analysis of the 2014 to 2016 data, the research team found that the prevalence of high-risk HPV in a sample of 5,749 young adult women living in Puerto Rico was 35.2% (n = 2,022). HPV prevalence observed in the years of the study remained somewhat stable (39% in 2014, 34% in 2015, and 32% in 2016). From 2014 and 2016, there was a 7.4% decrease in the prevalence. Although information regarding the prevalence of high-risk HPV is very limited in Puerto Rico, these results coincide with those of Méndez and colleagues [16], who studied HPV infection in a small population of 18- to 34-year-old women living in Puerto Rico; this group reported a 30.4% prevalence (n = 28) of high-risk HPV infection. Past research on the prevalence of HPV in Hispanics and Blacks between the ages of 14 to 59 residing in the U.S. reported a prevalence of 44.8% (n = 189) for women aged 20 to 24 and 27.4% (n = 174) for those aged 25 to 29 years old [13]. Similarly, there was a prevalence of 40.6% for women aged 21 to 23 and of 32.8% for 24 to 29-year-olds. These results corroborate past findings that the highest prevalence of high-risk HPV was observed in women aged 20 to 24 years old.

In the study, the genotypes most highly associated with cervical cancer (such as 31, 33, 35, 39, 45, 51, 52, 56, 58, 59, 66, and 68) had a combined prevalence of 85.41% (n = 1,727). Of the most aggressive HPV genotypes, the HPV 16 had the highest prevalence in 24- to 26-year-old women, followed by the HPV 18 in 27- to 29-year-old women. These findings correlate with Méndez and colleagues’ study where HPV 16 was the most prevalent of the high-risk genotypes, reported in 10.9% of a population of Puerto Rican women aged 18 to 34 (n-10) [16]. Finally, 15 women (0.74%) were co-infected with genotypes 16 and 18, most frequently occurring between 27 and 29 years of age (10 women).

Women infected with HPV genotypes 16 and 18 are at the greatest risk of developing high-grade lesions, which have a high probability of being persistent over time [19]. It is well understood that high-risk HPV infections are common and are associated with elevated cervical cancer risk. However, some studies suggest that approximately 90% of young women infected with a high-risk strain of HPV will clear the virus within two years [20]. Results indicated that over half (n = 100; 51.0%) of the women with two positive results in the study period tested positive for a strain of high-risk HPV. It was unclear whether an individual’s infection during this period was persistent (meaning a sustained infection consisting of a single, unvarying HPV strain) or if it was a second infection consisting of a different type of HPV instead. Past research demonstrates that having this virus for two years leaves the infected patient at a higher risk of developing malignant intraepithelial lesions [19]. Contrary to those studies claiming that women infected with high-risk HPV can (and do) eliminate the virus in a period of approximately 16.3 months, the research team found that only 49.7% (n = 108) of the women in the sample population with an initial HPV-positive result experienced a clearance of their HPV infection (defined as a negative result after an initial positive result) [21, 22]. However, it is unclear whether any of these women were treated for their infections, nor if any of them tested positive for HPV at any time prior to the years analyzed.

This is the largest study to date reporting the prevalence of high-risk HPV in young women residing in Puerto Rico, and results can serve as a basis for future epidemiological studies that may contribute to the development of better strategies for the prevention and early detection of cervical cancer in young women. This strength is tempered by a few limitations. The three-year study period was not sufficient to determine whether women with more than one HPV-positive result experienced a clearance of their infections. Accordingly, the team could not identify those women in whom the virus cleared after the time of the study (e.g., three or four years after having become infected). Also, a relatively small number of women had two or three positive test results, which limited the ability to ascertain clearance of infections. The team did not ask or identify which women previously received or did not receive the HPV vaccine. Finally, the retrospective nature of this analysis precludes the research team from determining whether a given patient’s consecutive positive results were due to the persistence of an initial HPV infection or a second infection that occurred after the clearance of the first. Of the patients whose first test was negative, 14.3% (n = 32) were found to be positive at the time of their second test; 86.0% (n = 191) remained negative.

## 5. Conclusion

In conclusion, during the period of the study, there was an increased general prevalence (35.17%) of high-risk HPV in the 21 to 29 age group, with the highest specific prevalence (40.6%) found in the younger subpopulation of women who were 21 to 23 years old. The results are like those that reported the prevalence of high-risk HPV infection in young women in the U.S. and Puerto Rico. Future studies should aim to evaluate high risk HPV infections by including a larger sample size of women of reproductive age, taking into consideration the impact of the HPV vaccine and the entry requirements for schools, to reduce cervical cancer in this population. These authors also recommend including cervical cytology results to correlate the presence of intraepithelial lesions with the HPV results of a given patient. Furthermore, there is a need for an evaluation of how Puerto Rican women’s’ access to HPV and cervical cancer screening tests has been affected due to natural disasters such as Hurricane Maria, recent earthquakes, and the COVID-19 pandemic.

## Figures and Tables

**Table 1:** Type Specific Seroprevalence for oncogenic HPV types

HPV Type	Number of Women (N)	Seroprevalence (%)
16	202	9.99
18	78	3.85
16/18	15	0.74
Other high-risk HPV (31, 33, 35, 39, 45, 51, 52, 56, 58, 59, 66, 68)	1,727	85.41

**Table 2:** Analysis of women with two or more HPV consecutive results from 2014 to 2016

Initial HPV Result	Second Result HPV Positive	Second Result HPV Negative
Positive (N = 196)	100 (51.0%)	96 (48.9%)
Negative (N = 223)	32 (14.3%)	192(86.0%)

## Data Availability

Ponce Health Sciences University (PHSU) and Ponce Research Institute (PRI) are committed to upholding the NIH Policy on Sharing of Research Data. The data information for this analysis is available in the Southern Pathology Services, Inc., private laboratory. The project administrators at this pathology laboratory are Dr. Adalberto Mendoza, and Dr. Rosa Vélez which are also authors in this study. Published research data will not include information that can identify the human subject participants, in accordance with IRB policies and will be devoid of private health identifiers (PHI) as defined by the Health Insurance Portability and Accountability Act (HIPM) of 1996. Data (without personal identifiers) will be stored and shared electronically if necessary.

## Abbreviations

HPV: Human Papilloma Virus
ASC: American Society of Cancer
ASCCP’s: American Society for Colposcopy and Cervical Pathology
SPS: Southern Pathology Services, Inc
UPR-MSC: University of Puerto Rico, Medical Sciences Campus
IRB: Institutional Review Board
